# Impact of Advanced Maternal Age on Laboratory Markers in Preeclamptic Pregnancies: A Cross‐Sectional Study

**DOI:** 10.1155/ogi/8830801

**Published:** 2026-05-30

**Authors:** Subanova Gulzhamal Arstanalievna, Priti Singh, Salman Khan, Subanova Nargiza Abdivalievna, Yrysbaev Erzamat Yrysbaevich, Yrysbaev Azamat Yrysbaevich

**Affiliations:** ^1^ International Medical Faculty, Department of Obstetrics and Gynecology, Osh State University, Osh, Kyrgyzstan; ^2^ Department of Nutritional, Biochemical, and Molecular Sciences, American University School of Medicine Aruba, Aruba Campus, Oranjestad, Aruba; ^3^ Department of Pathological Processes and Therapeutics, American University School of Medicine Aruba, Aruba Campus, Oranjestad, Aruba; ^4^ International Medical Faculty, Research at Osh State University, Osh, Kyrgyzstan; ^5^ Department of Obstetrics and Gynecology, No. 2 Kyrgyz State Medical Academy Named After I. K. Akhunbaev, Bishkek, Kyrgyzstan; ^6^ International Medical Faculty, Department of Medicine, Osh State University, Osh, Kyrgyzstan; ^7^ Department of Therapeutic Disciplines, Higher School of Medicine, Salymbekov University, Bishkek, Kyrgyzstan

**Keywords:** activated partial thromboplastin time, age, hemoglobin, over 35 years, platelets, preeclampsia, prenatal fibrinogen, PT, PTI, soluble fibrin monomer complexes

## Abstract

**Background and Aims:**

Preeclampsia is a major cause of maternal and fetal morbidity worldwide. Advanced maternal age is considered an important risk factor for adverse pregnancy outcomes. This study aimed to compare hematological, coagulation, and renal laboratory parameters between preeclamptic women aged < 35 years and ≥ 35 years in order to determine the impact of advanced maternal age on disease severity.

**Methods:**

A cross‐sectional study was conducted at Osh Interregional Clinical Hospital, Kyrgyzstan. 67 Pregnant women were included and categorized into severe preeclampsia (*n* = 48), moderate preeclampsia (*n* = 12), and normotensive controls (*n* = 7). Participants were divided into two age groups: under 35 years and ≥ 35 years. Hematological parameters (hemoglobin, RBC count, and platelets), coagulation markers (prothrombin time, INR, and fibrinogen levels), and renal function tests (serum creatinine and urinary protein levels) were analyzed.

**Results:**

Women with severe preeclampsia showed significantly lower hemoglobin levels (< 35 years: 112.15 g/L, > 35 years: 123 g/L, *p* < 0.05) and platelet counts. Prolonged PT (13.59 s in < 35 years vs. 12.73 s in > 35 years, *p* < 0.05) and reduced INR values (0.93 in > 35 years vs. 0.96 in < 35 years, *p* < 0.005) indicated hypercoagulability. Fibrinogen levels were elevated in severe preeclampsia (4.9 g/L in < 35 years vs. 4.5 g/L in > 35 years, *p* < 0.05). Renal dysfunction was evident with increased serum creatinine levels and significant proteinuria (*p* < 0.05).

**Conclusions:**

Advanced maternal age significantly influences hematological and coagulation abnormalities in preeclampsia, contributing to a hypercoagulable state and renal impairment. Regular laboratory monitoring, early screening, and individualized management strategies are crucial to mitigating adverse maternal and fetal outcomes.

**Clinical Significance:**

This study highlights the critical role of laboratory markers in detecting and managing preeclampsia, particularly in older pregnant women who are at higher risk of severe complications. The findings emphasize the necessity of early intervention strategies, such as frequent monitoring of coagulation and renal function, to reduce maternal morbidity and mortality. Understanding the hematological and biochemical variations associated with advanced maternal age in preeclampsia allows clinicians to implement personalized treatment plans aimed at improving both maternal and neonatal outcomes. These results also support the inclusion of laboratory marker analysis in routine prenatal care, enhancing the prediction and prevention of severe preeclampsia‐related complications.

## 1. Introduction

Preeclampsia is one of the most serious complications of pregnancy, affecting approximately 5%–8% of pregnant women worldwide. It is responsible for over 50,000 maternal deaths annually across different regions [1]. According to the Ministry of Health of the Kyrgyz Republic, hypertensive disorders during pregnancy ranked as the leading cause of maternal mortality in 2021, accounting for 21.1% of cases, compared to 7.5% in 2020 [2]. Preeclampsia is characterized by the development of hypertension, proteinuria, and dysfunction of multiple organ systems, posing a significant threat to both maternal and fetal health.

Maternal age is a well‐established risk factor for preeclampsia. Women over the age of 35 often experience more severe disease progression due to underlying comorbidities and vascular changes, whereas younger patients typically develop the condition in association with newly emerging disorders [3].

In recent decades, anemia has emerged as a global “noncommunicable epidemic,” affecting both resource‐limited and developed countries. According to the World Health Organization (WHO), iron deficiency affects more than 2 billion people worldwide, meaning approximately one in every three to four individuals. Currently, nearly 0.5 billion women of reproductive age, including 50–60 million pregnant women, are diagnosed with anemia [4]. Hemoglobin (Hb), along with the oxygen it transports, plays a crucial role in ensuring both maternal and fetal well‐being. Anemia during pregnancy results from an imbalance between the body’s high iron demands and its inadequate intake. Since iron is essential for tissue oxygenation, its deficiency leads to hypoxia, which contributes to anemia, reduced serum iron levels, and diminished iron reserves in the bone marrow.

The severity of anemia influences its complications, ranging from mild to severe. According to Radzinsky et al. [5], the prevalence of anemia in women varies from 5.4% in developed nations to as high as 80% in developing countries. Globally, anemia remains a major public health concern, affecting approximately 529 million women of reproductive age, including 38% of all pregnant women. It is a significant contributor to maternal mortality and adverse birth outcomes in both high‐ and low‐income countries. Studies have shown that low maternal Hb levels (< 110 g/L) are associated with poor birth outcomes, including low birth weight, preterm birth, small‐for‐gestational‐age (SGA) infants, stillbirth, perinatal and neonatal mortality, and maternal complications such as postpartum hemorrhage and preeclampsia [6].

The prevalence of anemia among pregnant women varies widely, ranging from 25% to 50% globally. In developing countries, it is estimated to affect 35%–75% of pregnant women, compared to 18%–20% in developed nations. In the Russian Federation, the incidence of anemia in pregnancy is reported to be approximately 32%. Among women from Central Asian countries, iron deficiency anemia (IDA) is particularly prevalent, with rates as high as 60%–90% in Uzbekistan. Notably, among maternal deaths in this region, IDA was present in 97%‐98% of cases. Additionally, hypertensive disorders, including preeclampsia, are reported in 32%–45% of pregnant women with IDA [7]. In the Kyrgyz Republic, severe preeclampsia and its complications, such as eclampsia, HELLP syndrome, and acute respiratory distress syndrome (ARDS), consistently rank as the second leading cause of maternal mortality, following obstetric hemorrhage [8].

The hemostatic system plays a critical role in maintaining normal physiological function within the fetoplacental system. Pregnancy is associated with significant alterations in coagulation factors, generally leading to an increased coagulation potential. For instance, fibrinogen levels begin to rise in the third month of pregnancy and peak at term, ensuring adequate hemostasis after placental separation. Concurrently, factor XIII activity decreases by 40%–50% by the end of the third trimester. Both of these factors are crucial for implantation and placental development. Any deviations in fibrinogen levels—whether elevated or reduced—can result in fetoplacental insufficiency, fetal hypoxia, intrauterine growth restriction, and, in severe cases, intrauterine fetal demise [9].

The Osh region of Kyrgyzstan is characterized by a predominantly rural population, limited access to specialized maternal healthcare services, and a relatively high prevalence of pregnancy‐related anemia and hypertensive disorders. These socioeconomic and healthcare factors make the study of preeclampsia particularly relevant in this region.

Given these unique socio‐economic and geographical conditions, studying the impact of maternal age on laboratory parameters in preeclamptic pregnancies is particularly relevant. A better understanding of these pathophysiological mechanisms will aid in refining diagnostic, prognostic, and treatment strategies. This study aims to conduct a comparative analysis of laboratory parameters in pregnant women with preeclampsia across different age groups (≤ 35 years vs. > 35 years) in the Osh region, with the objective of identifying age‐related variations in disease progression.

## 2. Materials and Methods

This study was conducted at the Osh Interregional Clinical Hospital (OIRCH) in the Osh region, Kyrgyzstan. A total of 67 pregnant women were included and categorized into three groups: severe preeclampsia (*n* = 48), moderate preeclampsia (*n* = 12), and normotensive controls (*n* = 7). The control group consisted of pregnant women without hypertension or clinical signs of preeclampsia. Among women with severe preeclampsia (*n* = 48), 13 were aged < 35 years and 31 were aged ≥ 35 years.

Preeclampsia severity was classified according to the American College of Obstetricians and Gynecologists (ACOG) guidelines. Severe preeclampsia was defined as blood pressure ≥ 160/110 mmHg with evidence of end‐organ involvement, while moderate preeclampsia was defined as blood pressure ≥ 140/90 mmHg with proteinuria but without severe features.

### 2.1. Inclusion Criteria

• Singleton pregnancy
• Gestational age of ≥ 20 weeks
• Diagnosis of preeclampsia based on ACOG guidelines [10].

### 2.2. Exclusion Criteria

• Chronic hypertension or preexisting renal disease
• Gestational diabetes mellitus
• Autoimmune or hematologic disorders
• Multiple pregnancies

### 2.3. Laboratory Assessments

Venous blood and urine samples were collected at the time of admission for the following analyses:

• Hematological parameters: Red blood cell (RBC) count, Hb, hematocrit, platelet count, and erythrocyte sedimentation rate (ESR).
• Coagulation profile: Prothrombin time (PT), prothrombin index (PTI), international normalized ratio (INR), fibrinogen, activated partial thromboplastin time (aPTT), and soluble fibrin monomer complexes (SFMC).
• Renal function tests: Serum creatinine and urinary protein levels.

### 2.4. Statistical Analysis

Statistical analysis was performed using IBM SPSS Statistics version 26.0 (IBM Corp., Armonk, NY, USA). Data distribution was assessed using the Shapiro–Wilk test. Normally distributed variables were analyzed using the Student’s *t*‐test, whereas nonparametric variables were compared using the Mann–Whitney *U* test and Fisher’s exact. Pearson correlation analysis was performed to evaluate associations between coagulation parameters and preeclampsia severity. All tests were two‐tailed, and a *p* value < 0.05 was considered statistically significant.

### 2.5. Ethical Approval

The University Ethical Committee at the International Medical Faculty, Osh State University, approved the study for OIRCH of Osh, Kyrgyzstan. Written informed consent was obtained from all participants before enrolment.

## 3. Results

The laboratory findings revealed statistically significant differences in hematological, coagulation, and renal function parameters between preeclamptic and normotensive women, as well as between age groups (see Tables 1–3). A comparative analysis of early‐ and late‐onset preeclampsia is presented in Table 4. Early‐onset preeclampsia was associated with significantly lower gestational age at delivery, lower birth weight, higher blood pressure values, and higher proteinuria compared to late‐onset preeclampsia (*p* < 0.001). The Pearson correlation test in this study helps to identify important relationships between coagulation markers and preeclampsia severity, providing insights into the hypercoagulable state observed in severe cases, which could be useful for early prediction and management of coagulation‐related complications in preeclamptic pregnancies (see Table 5).

**TABLE 1:** Laboratory parameters with preeclampsia in pregnant women.

	Severe preeclampsia with proteinuria/creatinine ratio ≥ 30	Severe preeclampsia with proteinuria/creatinine ratio ≤ 30	Moderate preeclampsia	Without preeclampsia	*p*
	*N* = 31	*N* = 16	*N* = 14	*N* = 6	
Red blood cells before birth	4.02 ± 0.19	4.03 ± 0.22	3.96 ± 0.24	3.93 ± 0.15	*p* < 0.05
Hemoglobin in the blood before birth	112.74 ± 6.25	108.31 ± 9.73	107.92 ± 7.62	113.83 ± 8.76	*p* < 0.05
Hematocrit	31.48 ± 1.69	30.88 ± 2.17	32.4 ± 1.83	33.2 ± 5.38	*p* > 0.05
Platelets	**240.16 ± 35.96**	**216.81 ± 46.71**	**259.46 ± 30.32**	158.66 ± 20.83	*p* < 0.05
ESR	29.56 ± 3.47	31.18 ± 4.76	30.0 ± 13.52	29.33 ± 24.13	*p* > 0.05
Prothrombin time	**13.25 ± 0.54**	**13.18 ± 0.37**	12.9 ± 0.8	12.4 ± 0.37	*p* < 0.05
Prothrombin index	**103.43 ± 3.59**	**103.96 ± 3.82**	98.13 ± 5.42	99.15 ± 4.24	*p* < 0.005
International normalized ratio	0.95 ± 0.04	0.94 ± 0.04	0.99 ± 0.05	0.99 ± 0.05	*p* < 0.005
Fibrinogen before birth	4.78 ± 0.33	5.05 ± 0.36	4.69 ± 0.33	4.65 ± 0.93	*p* < 0.05
Activated partial thromboplastin time	30.8 ± 2.1	30.38 ± 1.5	26.95 ± 4.37	31.02 ± 2.68	*p* < 0.05
Soluble fibrin monomer complexes	7.23 ± 0.62	6.86 ± 0.62	9.33 ± 3.18	6.25 ± 1.38	*p* > 0.05

*Note:* Bold values indicate statistically significant differences between groups (*p* < 0.05).

**TABLE 2:** Laboratory parameters in women with severe preeclampsia stratified by maternal age (< 35 years, *n* = 13; ≥ 35 years, *n* = 31).

	Group of pregnant women by age			
	Up to 35 years		After 35 years	
	Mean	Standard error of mean	Mean	Standard error of mean
Red blood cells before birth	3.97	0.06	4.09	0.12
Hemoglobin before birth	109.71	2.19	113.47	4.09
Hemotocrit	31.57	0.61	31.68	0.99
Platelet	239.24	14.07	222.06	15.03
Erythrocyte sedimentation rate	29.45	1.88	31.59	1.92
Prothrombin time	13.16	0.19	12.91	0.19
Prothrombin index	101.67	1.36	102.96	1.80
International normalized ratio	0.97	0.01	0.95	0.02
Fibrinogen before birth	4.90	0.12	4.61	0.14
Activated partial thromboplastin time	30.60	0.95	29.89	1.18
Soluble fibrin monomer complexes	7.32	0.25	7.98	1.10

**TABLE 3:** Maternal and clinical characteristics of study groups.

Variable	Severe < 35 (*n* = 13)	Severe ≥ 35 (*n* = 31)	Moderate (*n* = 13)	Control (*n* = 7)
Maternal age	26.5 [24.2–28.8]	38.0 [36.5–39.5]	30.5 [28.8–32.2]	29.0 [27.0–34.5]
Gestational age at delivery	35.5 [34.2–36.8]	37.0 [36.5–38.0]	38.0 [37.0–38.0]	38.0 [38.0–39.0]
Birth weight	2600 [2175–2875]	2700 [2400–2850]	3150 [2975–3225]	3300 [2900–3410]
Systolic blood pressure	166.5 [160.5–173.8]	165.0 [161.0–169.0]	144.5 [141.5–148.5]	110.0 [100.0–110.0]
Diastolic blood pressure	106.5 [100.5–110.0]	105.0 [101.0–106.5]	90.0 [89.5–92.5]	70.0 [70.0–70.0]
Proteinuria	3.5 [3.1–4.0]	3.2 [3.0–3.6]	1.1 [1.0–1.3]	0.0 [0.0–0.0]

**TABLE 4:** Comparison of clinical and laboratory parameters between early‐onset (< 34 weeks) and late‐onset (≥ 34 weeks) preeclampsia.

Variable	Early‐onset (< 34 weeks), *n* = 14, median [IQR]	Late‐onset (≥ 34 weeks), *n* = 46, median [IQR]	*p* value
Maternal age (years)	28.0 [26.0–30.0]	32.0 [30.0–35.0]	**0.0006**
Gestational age at delivery (weeks)	31.0 [30.0–32.5]	37.0 [36.0–38.3]	**< 0.001**
Birth weight (grams)	1650 [1500–1850]	3050 [2850–3250]	**< 0.001**
Maximum systolic BP (mmHg)	175 [170–180]	160 [158–162]	**< 0.001**
Maximum diastolic BP (mmHg)	113 [110–116]	100 [98–102]	**< 0.001**
Proteinuria (g/L)	4.5 [4.0–5.0]	2.7 [2.2–3.2]	**< 0.001**
Proportion with severe preeclampsia (%)	11/14 (79%)	29/46 (63%)	0.347
Proportion with moderate preeclampsia	3/14 (21%)	17/46 (37%)	0.347

*Note:* *p*‐value of <0.05 is considered statistically significant. Interpretation of the bold *p*‐values. Maternal age 0.0006 (significant difference). Gestational age at delivery <0.001 (highly significant). Birth weight <0.001 (highly significant). Maximum systolic BP <0.001 (highly significant). Maximum diastolic BP <0.001 (highly significant) Proteinuria <0.001 (highly significant).

**TABLE 5:** Correlation between prothrombin index and international normalized ratio (INR).

Parameter	Pearson correlation coefficient (*r*)	*p* value	Interpretation
Prothrombin index	**0.308**	**0.012**	Significant *positive correlation* with preeclampsia severity. As the prothrombin index increases, preeclampsia may worsen.
International normalized ratio (INR)	**−0.245**	**0.048**	Significant *negative correlation* with preeclampsia severity. Lower INR may be linked to increased severity, indicating a hypercoagulable state.

*Note:* The bold values indicate statistically significant correlations between coagulation parameters and preeclampsia severity because the *p*‐values are < 0.05. Prothrombin index (*r*) 0.308; *p* value 0.012 (significant positive correlation) INR (*r*−) 0.245; *p* value 0.048 (significant negative correlation).

### 3.1. Hematological Alterations

◦ The mean Hb level was significantly lower in women < 35 years with severe preeclampsia (112.15 g/L) compared to those > 35 years (123 g/L) (*p* < 0.05).
◦ RBC count was lower in women with early severe preeclampsia (< 35 years: 3.45 × 10^12^/L, > 35 years: 4.3 × 10^12^/L), indicating anemic tendencies in younger patients.
◦ Platelet count showed a decline with increasing severity of preeclampsia, with the lowest levels in severe cases (243.4 × 10^9^/L in < 35 years; 247.3 × 10^9^/L in > 35 years).

### 3.2. Coagulation Profile

◦ PT was prolonged in severe preeclampsia, with a mean of 13.59 s in < 35 years and 12.73 s in > 35 years (*p* < 0.05).
◦ PTI was significantly higher in severe cases (101.57% in < 35 years vs. 105.33% in > 35 years, *p* < 0.005), reflecting increased thrombotic risk.
◦ INR was significantly lower in preeclamptic women > 35 years (0.93 vs. 0.96 in < 35 years, *p* < 0.005), suggesting a hypercoagulable state.
◦ Fibrinogen levels were markedly elevated in severe preeclampsia (4.9 g/L in < 35 years vs. 4.5 g/L in > 35 years, *p* < 0.05), consistent with increased coagulation activity.

### 3.3. Renal Function Markers

◦ Serum creatinine levels were significantly higher in severe preeclampsia, indicating impaired kidney function (*p* < 0.01).
◦ Urinary protein excretion was significantly elevated in preeclamptic women (*p* < 0.05), with higher values in severe cases correlating with disease severity.

### 3.4. Correlation Between Prothrombin Index and INR

◦ The positive correlation between prothrombin index and preeclampsia suggests that as preeclampsia progresses, coagulation activity increases, leading to a higher PTI.
◦ The negative correlation between INR and preeclampsia suggests that lower INR values (reflecting increased clotting tendency) are associated with more severe preeclampsia.

## 4. Discussion

Our study demonstrates significant alterations in hematological, coagulation, and renal function parameters in preeclamptic pregnancies, particularly in women aged ≥ 35 years. The findings align with prior research and provide critical insights into the pathophysiology and clinical implications of preeclampsia.

### 4.1. Hematological Alterations

Preeclampsia is frequently associated with anemia, reflected in our study by significantly lower Hb levels in younger women with severe preeclampsia (112.15 g/L in < 35 years vs. 123 g/L in > 35 years, *p* < 0.05). These results align with Young et al., who reported a strong association between maternal anemia and adverse pregnancy outcomes [6]. Anemia in preeclampsia is attributed to increased hemolysis and hemodilution, leading to chronic placental hypoxia [11].

The RBC count was significantly lower in early severe preeclampsia (< 35 years: 3.45 × 10^12^/L vs. > 35 years: 4.3 × 10^12^/L). Similar findings have been documented by Jahromi and Rafiee, indicating the role of erythropoietic suppression due to oxidative stress in severe preeclampsia [12].

Platelet counts also declined as preeclampsia severity increased (243.4 × 10^9^/L in < 35 years vs. 247.3 × 10^9^/L in > 35 years). Salvi et al. confirmed that platelet consumption in preeclampsia results from endothelial damage and increased thrombin activity [13].

### 4.2. Coagulation Profile Alterations

Our study revealed prolonged PT in severe preeclampsia, with mean values of 13.59 s in < 35 years and 12.73 s in > 35 years (*p* < 0.05). Elevated PT values indicate a delayed clotting response, consistent with findings by Han et al., who reported prolonged coagulation times in preeclamptic women [14].

The PTI was significantly higher in severe cases (101.57% in < 35 years vs. 105.33% in > 35 years, *p* < 0.005). Increased PTI suggests a hypercoagulable state, also reported in studies by Fatkullina et al. and Kontovazainitis et al. [15, 16].

The INR was significantly lower in women with preeclampsia > 35 years (0.93 vs. 0.96 in < 35 years, *p* < 0.005). A lower INR indicates increased coagulation activity, as confirmed by Jin et al., who reported decreased INR values in early preeclamptic pregnancies [17].

Elevated fibrinogen levels in severe preeclampsia (4.9 g/L in < 35 years vs. 4.5 g/L in > 35 years, *p* < 0.05) reinforce the hypercoagulable state. Manten et al. found significantly increased high‐molecular‐weight fibrinogen in preeclamptic women, contributing to increased thrombotic risk [18].

### 4.3. Renal Function Impairment

Renal dysfunction in preeclampsia is evident in our findings, with significantly elevated serum creatinine levels in severe cases (*p* < 0.01). Elevated creatinine indicates glomerular endotheliosis and impaired renal filtration [19]. The presence of significant proteinuria (*p* < 0.05) further supports glomerular damage, a hallmark of severe preeclampsia.

One limitation of this study is the relatively small sample size in the moderate preeclampsia and normotensive groups. These groups were included primarily for descriptive comparison, and therefore, no definitive conclusions were drawn from these subsets. The primary analysis and conclusions of this study focus on women with severe preeclampsia, where the sample size was larger and more statistically robust.

## 5. Clinical Implications

Given the increased thrombotic risk and renal impairment observed in preeclampsia, early screening and regular coagulation monitoring are crucial, particularly in older pregnant women. ACOG recommends low‐dose aspirin prophylaxis and Doppler ultrasound surveillance to mitigate risks in high‐risk pregnancies [10]. Our findings support early intervention strategies, including antihypertensive therapy and early delivery, when necessary, particularly in women aged ≥ 35 years [20].

## 6. Limitations

One limitation of this study is the relatively small sample size in the moderate preeclampsia and normotensive groups. Therefore, the primary analysis focused on women with severe preeclampsia, where the sample size was larger and allowed for more meaningful comparisons. Additionally, the single‐center design may limit the generalizability of the findings.

## 7. Conclusion

This study highlights significant hematological, coagulation, and renal dysfunction in preeclamptic pregnancies, with pronounced abnormalities in women over 35 years. Regular laboratory monitoring, early screening, and tailored management strategies are essential to reduce complications and improve maternal‐fetal outcomes.

## Author Contributions

Subanova Gulzhamal Arstanalievna: statistical analysis, contributed to the discussion section, interpreting the study’s findings in the context of existing literature. She was responsible for the study’s methodology. Priti Singh: conceived and designed the study, analyzed the data, and wrote the initial draft of the manuscript. Provided insights into clinical implications, focusing on maternal and neonatal outcomes. Assisted in the interpretation of results, particularly regarding the risks of post‐term pregnancy, and assisted in data collection. Salman Khan: contributed to the study’s design and data analysis. Assisted in interpreting the results, particularly the risk factors and management strategies. Revised and critically reviewed the manuscript for intellectual content and supervised the overall research project. Subanova Nargiza Abdivalievna: contributed to data collection from the Osh Interregional Clinical Hospital, ensuring the accuracy of the medical records and demographic data. Yrysbaev Erzamat Yrysbaevich: contributed to the data collection and helped in the analysis of labor and delivery details, including the use of induction methods like Misoprostol. Yrysbaev Azamat Yrysbaevich: assisted with the statistical analysis and literature review.

## Funding

This research did not receive any grant from any funding agency.

## Disclosure

Salman Khan affirms that this manuscript is an honest, accurate, and transparent account of the study being reported; that no important aspects of the study have been omitted; and that any discrepancies from the study as originally planned have been explained.

## Conflicts of Interest

The authors declare no conflicts of interest.

## Data Availability

The data supporting the findings of this study are available within the article and its supporting information.
